# Pioglitazone attenuates kidney injury in an experimental model of gentamicin-induced nephrotoxicity in rats

**DOI:** 10.1038/s41598-019-49835-1

**Published:** 2019-09-23

**Authors:** Branislava Medić, Marko Stojanović, Branislav Rovčanin, Dušan Kekić, Sanja Radojević Škodrić, Gordana Basta Jovanović, Katarina Savić Vujović, Nevena Divac, Radan Stojanović, Miroslav Radenković, Milica Prostran

**Affiliations:** 1 Department of Pharmacology, Clinical Pharmacology and Toxicology, Faculty of Medicine, University of Belgrade, Belgrade, 11000 Serbia; 2 Centre for Endocrine Surgery, Clinical Centre of Serbia, Faculty of Medicine, University of Belgrade, Belgrade, 11000 Serbia; 3 Institute for Microbiology and Immunology, Faculty of Medicine, University of Belgrade, Belgrade, 11000 Serbia; 4 Department of Pathology, Faculty of Medicine, University of Belgrade, Belgrade, 11000 Serbia

**Keywords:** Immunochemistry, Preclinical research

## Abstract

Gentamicin, belonging to the aminoglycosides, possesses the greatest nephrotoxic effect of all other antibiotics from this group. On the other hand, pioglitazone, which represents peroxisome proliferator-activated receptor γ (PPARγ) agonist recently showed antiinflamatory, antioxidative effects, amelioration of endothelial dysfunction etc. Therefore, the goal of our study was to investigate the effects of pioglitazone on kidney injury in an experimental model of gentamicin-induced nephrotoxicity in rats. These effects were observed by following values of biochemical (serum urea and creatinine) parametars, total histological kidney score, urine level of kidney injury molecule-1 (KIM-1) and neutrophil gelatinase-associated lipocalin (NGAL) as well as parametars of oxidative stress (malondialdehyde, superoxide dismutase, catalase, total oxidant status, total antioxidant status, oxidative stress index and advanced oxidation protein products). It seems that pioglitazone protects the injured rat kidney in a U-shaped manner. Medium dose of pioglitazone (1 mg/kg, i.p.) was protective regarding biochemical (serum urea and creatinine), total histological score and the values of kidney injury molecule-1 (KIM-1) (P < 0.05 vs. control group, i.e. rats injected with gentamicin only). This finding could be of great importance for the wider use of aminoglycosides, with therapy that would reduce the occurrence of serious adverse effects, such as nephrotoxicity and acute renal failure.

## Introduction

Aminoglycosides are a group of antibiotics, which are used for treatment of serious infections worldwide. However, their adverse effects, such as ototoxicity, nephrotoxicity, neuromuscular blockade and allergic skin reactions are still a topic of discussion^1^. Gentamicin, belonging to the aminoglycosides has the greatest nephrotoxic effect of all other antibiotics from this group. A recent study has shown that even one dose of this agent can lead to acute kidney injury^2^. Nephrotoxicity induced by gentamicin represents a complex phenomenon which is characterized by an increase in serum creatinine and urea levels, as well as severe proximal renal tubular necrosis^3^. The exact mechanism of gentamicin-induced renal damage has not been fully understood, but it seems that generation of reactive oxygen species (ROS) and inflammation have a crucial role in pathogenesis of kidney injury. For this reason, drugs with promising antiinflamatory, antioxidative and nephroprotective properties were in focus of examination in numerous preclinical and clinical studies in the past decade^4,5^.

Peroxisome proliferator-activated receptor-γ (PPAR-γ) receptors belong to the nuclear steroid hormone receptors. Specifically, PPARγ activation induces nitric oxide (NO) expression, therefore leading to vasodilation in the efferent arteriole in the glomerulus and prevention of vascular wall thickening in atherosclerosis^6^.

Thiazolidinediones (TZDs), which represent group of PPAR-γ agonists, have been previously shown to reduce interleukin 1 (IL-1), interleukin 6 (IL-6), and tumor necrosis factor-α (TNF-α), that are well known cytokines in inflammatory processes. It has been observed that TZDs also reduce plasma levels of C-reactive protein (CRP), which is a marker of vascular inflammation^7^. Pioglitazone, which belongs to the thiazolidinedione group of anti-diabetic agents, is a PPARγ agonist with revealed nephroprotective effects^2^. Recent study showed that pioglitazone reduced renal injury in experimental diabetic rats through its antiinflammatory actions, including inhibition of Nuclear factor kappa B (NF-κB) activation and macrophage infiltration in the kidney^8^. Additionally, nephroprotective effect of pioglitazone was confirmed in an experimental model of ischemia-reperfusion (I/R) kidney injury, using more specific injury markers such as kidney injure molecule 1 (KIM-1) and neutrophil gelatinase-associated lipocalin (NGAL)^9^.

The goal of our study was to investigate the effects of pioglitazone on kidney injury in an experimental model of gentamicin-induced nephrotoxicity in rats.

## Results

### Quantification of KIM-1 and NGAL in urine

**Figure 1:**
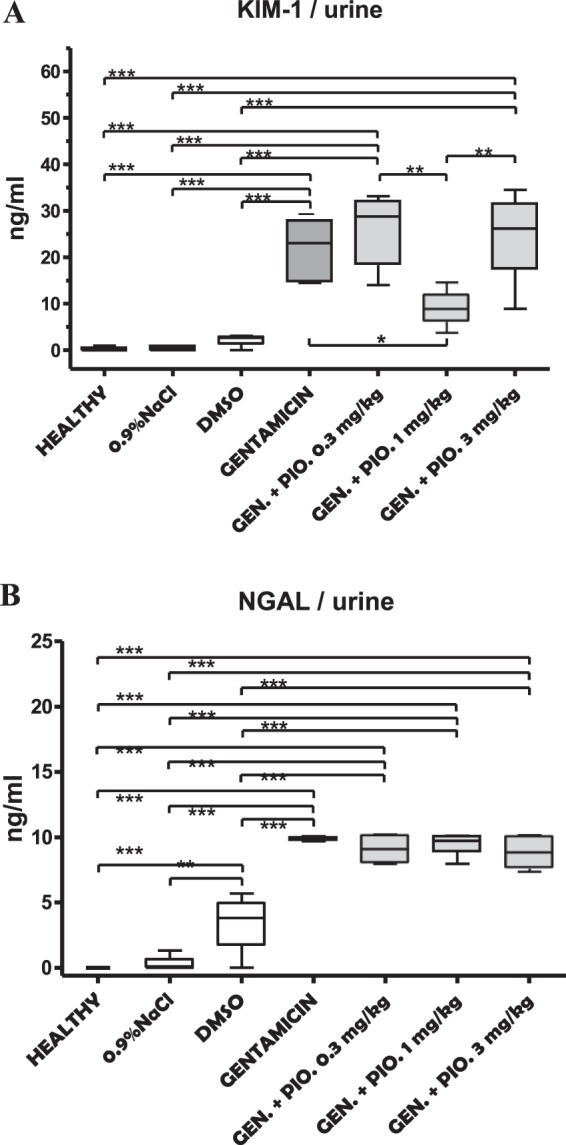
Box and whiskers plots showing urine levels of KIM-1 (**A**) and NGAL (**B**) in healthy, sham (0,9% NaCl), DMSO, gentamicin, and gentamicin plus pioglitazone in dose of 0.3, 1 and 3 mg/kg. The upper and the lower hinge of the boxes represent the interquartile range, the midline represents the median value, and the whiskers represent maximum and minimum values. *P < 0.05, **P < 0.01, ***P < 0.001.

The concentration of KIM-1 molecule in the animal urine was almost undetectable in the untreated (healthy group), sham group (0.9% NaCl) and the group treated with DMSO (Table 1, Fig. 1A). The administration of gentamicin has led to a statistically significant increase in urinary KIM-1 level (Table 1A, Fig. 1, P < 0.001). The simultaneous administration of gentamicin and pioglitazone, in a dose of 0.3 mg/kg and 3 mg/kg, also increased the concentration of KIM-1 in a similar manner as gentamicin alone did (Table 1, Fig. 1, P < 0.001). Interestingly, gentamicin given in a dose of 1 mg/kg significantly reduced the urine concentration of KIM-1 when compared to gentamicin alone (P < 0.05) and/or gentamicin plus piolitazone 0.3 mg/kg or 3 mg/kg (P < 0.01), creating a U-shaped curve (Table 1, Fig. 1A).

**Table 1:** The urinary level of KIM-1 (kidney injury molecule-1) and NGAL (neutrophil gelatinase-associated lipocalin) recorded in different experimental groups.

Group	KIM-1 (ng/ml)	NGAL (ng/ml)
Healthy	0.19 ± 0.40***	0.00 ± 0.00***^†††^
0.9% NaCl	0.35 ± 0.50***	0.22 ± 0.54***^†††^
DMSO	2.18 ± 1.44***	3.32 ± 2.41***
Gentamicin	21.95 ± 6.18	9.86 ± 0.16
Gentamicin + pioglitazone 0.3 mg/kg	25.99 ± 7.73	9.13 ± 1.15
Gentamicin + pioglitazone 1 mg/kg	9.04 ± 4.42*	9.52 ± 0.88
Gentamicin + pioglitazone 3 mg/kg	24.86 ± 9.54	8.89 ± 1.20

Each result represents the mean ± SD (n = 4–6). ***P < 0.001, *P < 0.05 compared to gentamicin group (*ANOVA* followed by the *Turkey* post hoc test). ^†††^P < 0.001 compared to DMSO group (*ANOVA* followed by the *Turkey* post hoc test).

The concentration of NGAL in a healthy and sham group was barely detectable (undetectable in the healthy group), while significantly increased in gentamicin and the groups with simultaneous administration of gentamicin and pioglitazone (Table 1, Fig. 1B). The administration of DMSO also increased the NGAL concentration in urine, when compared to healthy (p < 0.001) and sham group (p < 0.01). Still, this increase was lower than those observed after administration of gentamicin alone or in combination with pioglitazone (p < 0.001). When results were compared between gentamicin and the groups where gentamicin and pioglitazone were at the same time administrated, no difference was found (Fig. 1B).

### Measurement of urea, creatinine and Na^+^ level in animal serum

**Figure 2:**
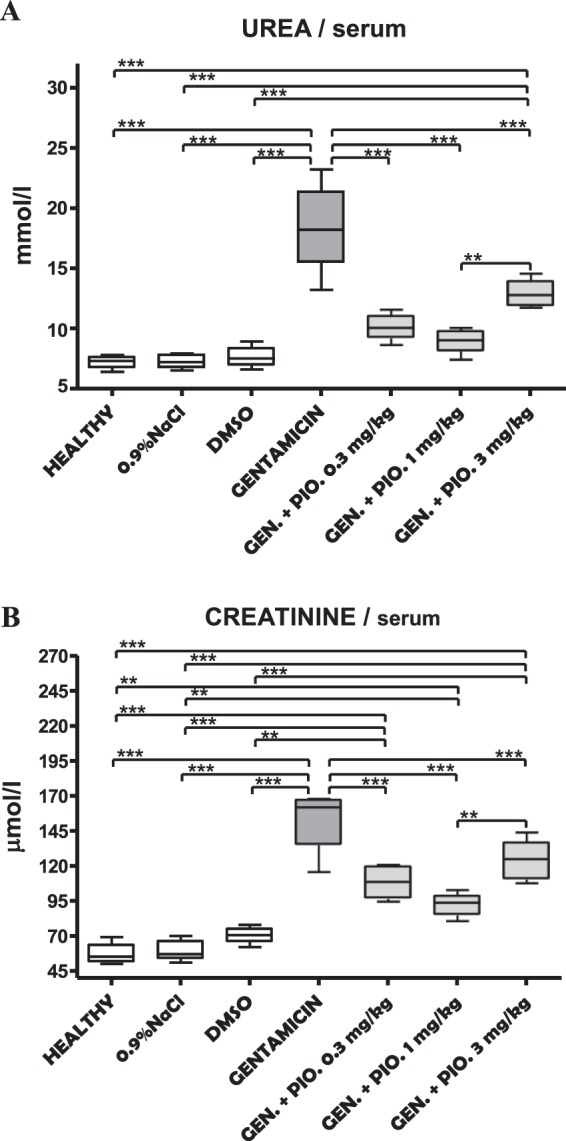
Box and whiskers plots showing serum levels of urea (**A**) and creatinine (**B**) in healthy, sham (0,9% NaCl), DMSO, gentamicin, and gentamicin plus pioglitazone in dose of 0.3, 1 and 3 mg/kg groups. The upper and the lower hinge of the boxes represent the interquartile range, the midline represents the median value, and the whiskers represent maximum and minimum values. **P < 0.01, ***P < 0.001.

The serum levels of urea were different among investigated animal groups. The highest concentrations were registered in the group of animals that received gentamicin. In other groups, urea levels were significantly reduced (Table 2, Fig. 2A, P < 0.001). Additionally, The group of animals receiving gentamicin + pioglitazone 3 mg/kg had a significantly higher urea level than a group of healthy, sham and DMSO (Table 2, Fig. 2A, P < 0.001), as well as animals that receive gentamicin and pioglitazone 1 mg/kg (Table 2, Fig. 2A, P < 0.01).

**Table 2:** The serum level of urea, creatinine and Na^+^ recorded in different experimental groups.

Group	UREA (mmol/l)	CREATININE (µmol/l)	Na^+^ (mmol/l)
Healthy	7.20 ± 0.53*** ^†††^	56.80 ± 7.46*** ^†††^	148.80 ± 1.64
0.9% NaCl	7.24 ± 0.57*** ^†††^	59.40 ± 7.30*** ^†††^	147.60 ± 2.07
DMSO	7.63 ± 0.98*** ^†††^	70.25 ± 6.65*** ^†††^	145.30 ± 3.20
Gentamicin	18.36 ± 3.59	153.40 ± 21.63	146.60 ± 4.04
Gentamicin + pioglitazone 0.3 mg/kg	10.05 ± 0.96***	108.60 ± 11.61***	148.50 ± 1.76
Gentamicin + pioglitazone 1 mg/kg	8.93 ± 0.89*** ^††^	92.40 ± 8.11*** ^††^	146.50 ± 3.15
Gentamicin + pioglitazone 3 mg/kg	12.82 ± 1.04 ***	124.20 ± 12.91***	148.50 ± 2.95

Each result represents the mean ± SD (n = 4–6). ***P < 0.001, **P < 0.01, *P < 0.05 compared to gentamicin group (*ANOVA* followed by the *Turkey* post hoc test). ^†††^P < 0.001 compared to gentamicin + pioglitazone 3 mg/kg (*ANOVA* followed by the *Turkey* post hoc test).

Similar as with urea, creatinine was also increased in gentamicin group. The creatinine level was significantly lower in the rest of experimental groups (Table 2, Fig. 2B, P < 0.001). Also, all the groups treated with gentamicin and pioglitazone combination had significantly higher levels of creatinine then healthy, shame and DMSO groups (Table 2, Fig. 2B, P < 0.001). Additionally, the creatinine values were significantly higher in a group of animals simultaneously treated with gentamicin and pioglitazone in highest dose (3 mg/kg) when compared to the group treated with gentamicin and pioglitazone in middle dose (1 mg/kg) (Table 2, Fig. 2B, P < 0.01).

The serum sodium level was unchanged after administration of gentamicin, which can be seen through almost identical sodium level between investigated groups (Table 2, Fig. 2A).

### Total histological score

**Figure 3:**
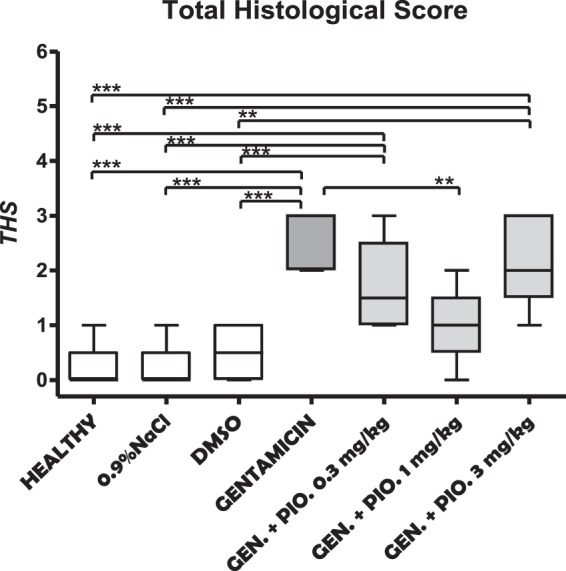
Box and whiskers plots showing total histological score (THS) in kidneys obtained from healthy, sham (0,9% NaCl), DMSO, gentamicin, and gentamicin plus pioglitazone in dose of 0.3, 1 and 3 mg/kg groups. The upper and the lower hinge of the boxes represent the interquartile range, the midline represents the median value, and the whiskers represent maximum and minimum values. **P < 0.01, ***P < 0.001.

The calculated THS, similar as in previous results, was highest in the animals treated with gentamicin only (Fig. 3). Gradation order of THS, from the highest to the lowest, looks like this gentamicin (2.60 ± 0.55), gentamicin + pioglitazone 3 mg/kg (2.20 ± 0.84), gentamicin + pioglitazone 0.3 mg/kg (1.67 ± 0.82), gentamicin + pioglitazone 1 mg/kg (1.00 ± 0.63), DMSO (0.50 ± 0.58), and at the end healthy (0.20 ± 0.45) and sham group (0.17 ± 0.41) (Fig. 3).

**Figure 4:**
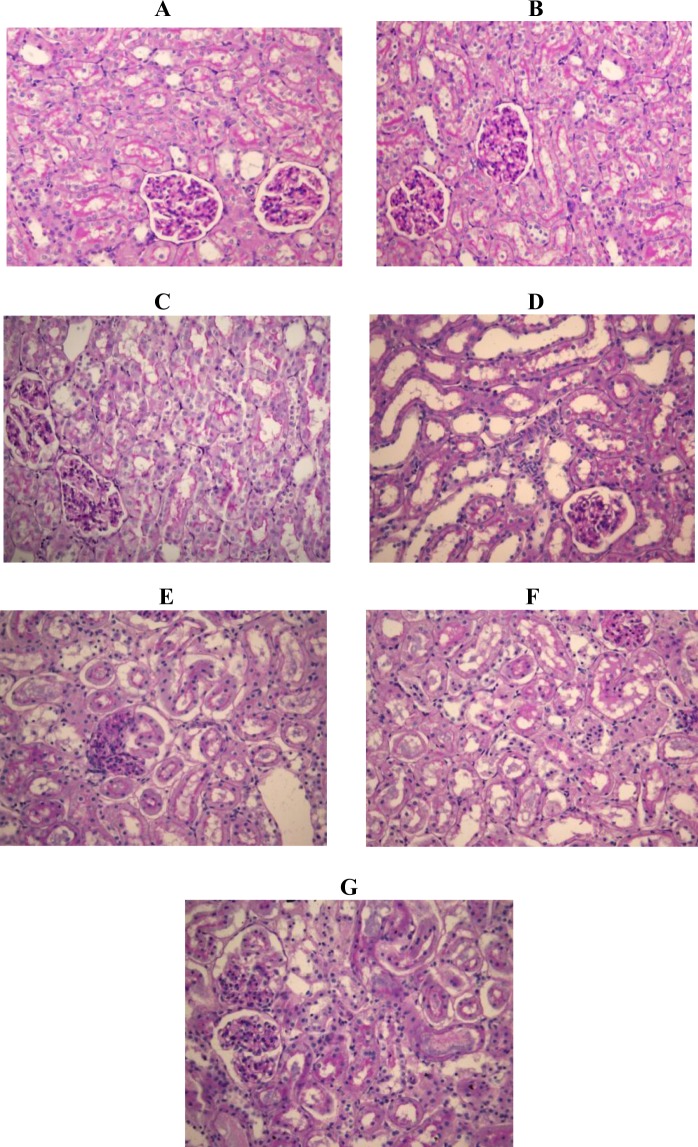
The effects of pioglitazone (0.3 mg/kg; 1 mg/kg; 3 mg/kg) on histological micrographs of renal tissues. Periodic acid–Schiff (PAS) stain coloring. Original magnification ×20. Figures were randomly chosen from the series of at least 6 experiments (Panels A–G). Panel A: Healthy animal - normal renal parenchyma (PAS staining). Panel B: Animals treated with DMSO only - normal renal parenchyma (PAS staining). Panel C: Animals treated with saline only - normal renal parenchyma (PAS staining). Panel D: Rats subjected to gentamicin-induced nephrotoxicity- marked kidney damage, interstitial edema diffusely present, proximal tubules show loss of brush border and lumen dilatation and loss of nuclei in some epithelial cells. Panel E: Rats subjected to gentamicin induced nephrotoxicity, treated with pioglitazone at dose of 0.3 mg/kg - moderate kidney damage, loss of brush border was observed in half of proximal tubules, in addition to dilatation of lumen and loss of nuclei in some epithelial cells. Panel F: Rats subjected to gentamicin induced nephrotoxicity, treated with pioglitazone at dose of 1 mg/kg –minimal to moderate kidney damage. Panel G: Rats subjected to gentamicin induced nephrotoxicity, treated with pioglitazone at 3 mg/kg – moderate to marked kidney damage, two thirds of proximal tubules show loss of brush border, dilatation of lumen and loss of nuclei in majority of epithelial cells (marked necrosis).

The effects of pioglitazone (0.3 mg/kg; 1 mg/kg; 3 mg/kg) on histological micrographs of renal tissues are presented in Fig. 4.

### Oxidative stress

Taken together, there was no significant difference in any examined oxidative stress parameter when healthy renal tissue and 0.9% NaCl controls were examined. The significant reduction of TOS and MDA concentration was observed in DMSO treated samples than in healthy and 0.9% NaCl control samples, which consequently lead toward the highly significant lowering of OSI values. It is evident that animals treated with gentamicin had markedly increased renal oxidative stress, based on the values of TOS, OSI, MDA, AOPP and SOD activity. Pioglitazone significantly reduces gentamicin induced renal oxidative stress in dose dependent pattern according to the OSI values. Additionally, highly significant differences in most of oxidative stress parameters were found when gentamicin + pioglitazone groups were compared with healthy, 0.9% NaCl and DMSO treated groups. As evident, addition of pioglitazone to the gentamicin treated animals does not affect renal MDA, AOPP, SOD and CAT values, when compared to the gentamicin treated animals (Table 3).

**Table 3:** The parameters of oxidative stress recorded in different experimental groups. Each result represents the mean ± SD (n = 4–6).

Group	*PROTEINS*	*TOS*	*TAS*	*OSI*	*MDA*	*AOPP*	*SOD*	*CAT*
Healthy	4.55 ± 2.61	1.37 ± 0.32	0.19 ± 0.04	7.02 ± 0.29	1.92 ± 0.50	6.55 ± 1.60	3.74 ± 0.33	48.97 ± 6.51
0.9% NaCl	5.61 ± 1.26	1.23 ± 0.42	0.17 ± 0.06	7.09 ± 0.31	1.92 ± 0.51	6.50 ± 1.42	3.75 ± 0.32	49.00 ± 6.55
DMSO	8.15 ± 1.14	0.40 ± 0.03^**††^	0.19 ± 0.03	2.16 ± 0.44	0.98 ± 0.21^**††^	3.26 ± 0.04	2.84 ± 0.16	35.82 ± 2.23
Gentamicin	7.59 ± 3.18	3.15 ± 0.42^***†††‡‡‡^	0.18 ± 0.08	21.99 ± 13.88^**††‡‡‡#&^	2.65 ± 0.31^**††‡‡‡^	7.15 ± 3.06^‡^	4.74 ± 0.89^*†‡‡‡^	57.48 ± 12.27^‡‡^
Gentamicin + pioglitazone 0.3 mg/kg	7.41 ± 4.02	2.95 ± 0.22^***†††‡‡‡^	0.23 ± 0.04	13.25 ± 3.05	2.74 ± 0.08^**††‡‡‡^	6.96 ± 2.40^‡^	4.95 ± 0.26^**††‡‡‡^	46.49 ± 4.85
Gentamicin + pioglitazone 1 mg/kg	5.90 ± 3.33	2.76 ± 0.32^***†††‡‡‡^	0.24 ± 0.03	11.37 ± 1.53	2.78 ± 0.15^**††‡‡‡^	8.40 ± 0.69^‡‡^	4.26 ± 0.66^‡‡^	52.04 ± 6.90^‡^
Gentamicin + pioglitazone 3 mg/kg	7.52 ± 4.19	2.49 ± 0.32^***†††‡‡‡^	0.25 ± 0.05	10.57 ± 2.80	2.67 ± 0.18^**††‡‡‡^	8.19 ± 0.76^‡‡^	4.81 ± 0.40^*†‡‡‡^	59.47 ± 6.18^‡‡‡^

*P < 0.05, **P < 0.01; ***P < 0.001 compared to healthy group (*ANOVA* followed by *Tukey* post hoc test);

^†^P < 0.05, ^††^P < 0.01; ^†††^P < 0.001 compared to 0.9% NaCl group (*ANOVA* followed by *Tukey* post hoc test);

^‡^P < 0.05, ^‡‡^P < 0.01, ^‡‡‡^P < 0.001 compared to DMSO group (*ANOVA* followed by *Tukey* post hoc test);

^#^P < 0.05 compared to GEN + PIO 1 mg/kg (*ANOVA* followed by *Tukey* post hoc test);

^&^P < 0.05 compared to GEN + PIO 3 mg/kg (*ANOVA* followed by *Tukey* post hoc test).

## Discussion

Our study showed that the administration of gentamicin at the dose of 100 mg/kg/day for 7 days i.p., successfully induced nephrotoxicity in all treated animals. This finding is in agreement with some previous reports^2,10,11^. Gentamicin-treated rats produced a significant increase in serum creatinine and urea concentrations, which was also previously revealed in the study performed by *Karahan et al*.^12^. Additionally, gentamicin induced tubular necrosis in rat’s kidneys, increased concentration of KIM-1 and NGAL as well as parameters of renal oxidative stress (TOS, OSI, MDA, AOPP and SOD activity).

The effects of pioglitazone on gentamicin-induced nephrotoxicity in our experimental model were different in relation to the administered dose. As described in detail above, our results showed a significant lower levels of serum urea and creatinine, as well as total histological score and KIM-1 after administration of medium dose of pioglitazone (1 mg/kg) with gentamicin, in comparison to sole administration of gentamicin or other concentrations of pioglitazone (0.3 and 3 mg/kg) creating a U-shaped curve. However, there was no statistically significant difference between gentamicin and the groups where gentamicin and pioglitazone were at the same time administrated regarding values of NGAL.

*Luo et al*. recently showed that the expressions of KIM-1 and NGAL changed prior to renal tubule damage and increase in serum creatinine and urea levels and correlated with the progressive histopathological alterations suggesting their usefulness for predicting gentamicin-induced acute kidney injury (AKI) *in vivo*^13,14^.

Additionally, our study showed that pioglitazone significantly reduces gentamicin-induced renal oxidative stress in dose dependent pattern according to the OSI values, although addition of pioglitazone to the gentamicin treated animals does not affect renal MDA, AOPP, SOD and CAT values, when compared to the gentamicin treated animals.

Some previous studies proved that pioglitazone can reduce oxidative stress by improving some antioxidant enzymes at the kidney level^15–19^. Beside this, *Sun* and his experimental group revealed that administration of pioglitazone increased the expression of SOD while decreased the level of MDA, implying the restoration of antioxidative capacity in the injured kidney^20^.

Several studies reported beneficial effects of PPAR-γ agonists on the kidney^21–24^. There are several possible explanation how pioglitazone can afford protection regarding kidney function. As has been previously shown, pioglitazone possesses antifibrotic and anti-inflammatory effects^25^. *Agrawal et al*. proved more information regarding antiinflammatory properties of pioglitazone. In their study pioglitazone enhanced glomerular filtration after injury by reducing of COX-2 expression^26^. Beside this, treatment with pioglitazone inhibited the activation of the NF-κB and MAPK inflammatory signaling pathways and reduced significantly the secretion of pro-inflammatory cytokines TNF-α, IL-1β, and IL-6^27^.

A meta-analysis performed by *Sarafidis et al*. concluded that TZDs possess some beneficial effects on kidney, such as reduction of glomerular hyperfiltration, prevention of intrarenal arteriolosclerosis, and prevention of glomerulosclerosis and tubulointerstitial fibrosis^7^. *Iglesias et al*. in their review also analyzed different, potentially protecting aspects TZDs on kidney function such as: hemodynamic effects (blood pressure reduction, vasodilatator effect on postglomerular efferent arteriole, increase NO production), metabolic effects (reduction of insulin resistance), antiinflammatory and antiproliferative effects (inhibition of cytokine production by macrophages, reduction of oxidative stress, reduction of polymorphonuclear infiltration) etc.^6^. Even though both of these studies did not focus on the specific gentamicin-induced nephrotoxicity treated with pioglitazone, they have demonstrated the ameliorating and beneficial effects of TZDs in general.

Some other paper confirmed beneficial effects of pioglitazone on kidney. In research performed by *Helmy et al*. pioglitazone in combination with fenofibrate led to the normalization of biochemical, morphological, inflammatory, oxidative, and apoptotic renal profiles via TNF-α inhibition in cisplatin-induced acute renal failure in rats^28^.

Beside of antiinflammatory and antioxidant properties, treatment with pioglitazone could exert vasculoprotective effects. It was demonstrated that pioglitazone increase eNOS and nNOS expression, normalized ratio between eNOS and iNOS and lead to regulation of glomerular blood flow and GFR by regulation of vascular tone of afferent arterioles^29,30^.

In addition of all the mentioned protective mechanisms, *Singh et al*. concluded that NMDA antagonism serves as one of the possible mechanisms in pioglitazone-mediated protection against renal ischemia reperfusion injury in rats^31^.

Our study showed for the first time that pioglitazone attenuates kidney injury in a U-shaped manner in an experimental model of gentamicin-induced nephrotoxicity in rats.

Future experiments should include a wider range of doses of pioglitazone and examine the effects of pioglitazone on some more specific markers of inflammation (such as NF-κB, TNF-α, IL-1, IL-6 etc.), some other markers of oxidative stress as well as novel markers of kidney injury (interleukin 18 *et al*.) in order to provide more accurate explanation regarding exact mechanisms of nephroprotective effects of pioglitazone in this experimental model.

Finally, it should be point out that his finding could be of great importance for the wider use of aminoglycosides, with therapy that would reduce the occurrence of serious adverse effects, such as nephrotoxicity and acute renal failure.

## Material and Methods

### Animals and ethical approval

Experiments were performed in adult, 2 months old, male Wistar rats (n = 40) weighing between 210 and 270 g. Before the beginning and during the experiments, animals were housed in polycarbonate cages (4 per cage). The living conditions were maintained constant at 12 h/12 h light/day cycle, a temperature of 24 ± 1 °C, and a humidity of 55 ± 10%. The animals had ad libitum access to food (pellets of standard rodent diet) and water (tap water).

The methodology used in our investigation was approved by the Ethical Commission for the Welfare Protection of Experimental Animals (Medical Faculty, University of Belgrade, 2017– 2379/02). Guidelines from the European Convention for the Protection of Vertebrate Animals Used for Experimental and other Scientific Purposes, Directive 2010/63/EU for animal experiments, the Guide for the Care and Use of Laboratory Animals and Good Laboratory Practice were fully followed.

### Experimental groups

Animals were randomly divided into one of seven groups. In first group were only healthy animals (n = 6); second was Sham group in which animals were given saline (0.9% NaCl) (n = 6); third group consisted of animals treated with DMSO (a substance used to dissolve pioglitazone) (n = 4); forth group received gentamicin (n = 6); while fifth (n = 6), sixth (n = 6) and seventh group (n = 6) received gentamicin and pioglitazone in a dose of 0.3, 1 and 3 mg/kg, respectively.

### Acute kidney damage induced by gentamicin

Commercially available gentamicin was used for induction of ARF. Gentamicin was used in a 100 mg/kg dose and it was administrated by intraperitoneal route (i.p.). The administration was repeated every day during a period of seven days. The process of administration was performed in the morning every day at the same time point.

### Sample collection

Sample collection was undertaken on a first day post last gentamicin administration. Before sample collection, animals were anesthetized with an intraperitoneal bolus injection of sodium thiopentone (Thiopental®, Nycomed Pharma, Unterschleibheim, Germany) in a dose of 120 mg/kg. Animals were allowed to stabilize and to enter deep anesthesia before the samples were taken. The depth of anesthesia was investigated by pinching the rat tail and the space between the toes with tweezers or by poking these areas with a needle. The absence of responding to both these stimuli was considered as established deep anesthesia.

After establishing of deep anesthesia, animals were open and bladder punction was performed in order to obtained urine for further investigation. Urine was placed in collecting vessels and stored at −80 °C until further use. The blood samples were obtained by heart puncture. The blood samples were for 2 h at the room temperature, in order to extract the serum and to allow samples to clot. After that, collecting vessels was centrifuged for 15 min at 1000 X g, which led to the final serum separation. Samples were stored at −80 °C for further future use. After cardiac punition, *post mortem*, both kidneys were harvested for histopathological investigation.

Immediately after the euthanasia, renal tissue specimens were collected from each animal and stored at −80 °C, until the further biochemical analyses were performed. After the samples were defreezed, renal tissue was mechanically homogenized in the sterile 0.05 M PBS (pH = 7.5) and then the total amount of proteins in each homogenate was determined by the method of Bradford^32^.

### Enzyme-linked immunosorbent assay (ELISA)

The urine level of kidney injury molecule-1 (KIM-1) and neutrophil gelatinase-associated lipocalin (NGAL) were detected using ELISA with kits produced by the Cusabio Biotech Co., Ltd. (Wuhan, China) and EIAab SCIENCE Cc., Ltd. (Wuhan, China) respectively. The provided instructions for those kits were fully applied. The results were detected with micro plate reader model DV 990 BV-6 (GDV, Gio Da Vita, Rome, Italy).

### Histopathology

Kidneys, post extraction, were placed in formalin and further processed to wax, sectioned into 5 µm thick slices and stained with Periodic acid-Schiff (PAS). For this purpose magnification x20 was used on an electronic light microscope (Leica DM LS 2, type 11020518016, Microsystems, Wetzlar, Germany).

After kidney preparation, samples were histologically evaluated and graded according to the predefined scoring system, which is based on a severity of kidney injury. A minimum of 10 fields for each kidney slide were examined and assigned for severity of changes. The scoring system was as follows: 0 - absent; 1 - mininal changes; 2 - moderate changes and 3 - marked changes^33^. In the end, all scores were auditioned in order to obtain a total histological score (THS). The histological samples analysis was blinded and performed by two experts (Department of Pathology, Faculty of Medicine, University of Belgrade).

### Oxidative stress

Lipid peroxidation as evidenced by the formation of thiobarbituric acid reactive substances (TBARS) was assayed in sera by the method of Varshney and Kale. The pink colored chromogen’s absorbance formed by the reaction of 2-thiobarbituric acid with the breakdown products of lipid peroxidation was read at 535 nm. Absorbances obtained from the standard curve were transferred to units of molar concentration of malondialdehyde (MDA) and expressed as mean micro molar concentration of MDA per mg of tissue proteins [μmol/mg proteins]^34^.

The superoxide dismutase (SOD) activity was determined according to the method of Sun and Zigman (1978) by measuring the absorbance change during autooxidation of adrenalin into adrenochrome at 340 nm. Commercial SOD from human erythrocytes (Sigma-Aldrich Darmastadt, Germany) was used as standard for evaluation of the total SOD activity. The SOD activity was expressed as unit of enzyme activity per mg of tissue proteins [U/mg proteins]^35^.

The catalase (CAT) activity was determined spectrophotometrically according to the method of Beers and Sizer (1952). The absorbance change during hydrogen peroxide breakdown by CAT was measured at 240 nm. Commercial CAT from human erythrocytes (Sigma-Aldrich) was used as standard for evaluation of the total CAT activity. The CAT activity was expressed as unit of enzyme activity per mg of tissue proteins [U/mg proteins]^36^.

The total oxidant status (TOS) was determined according to the method of Erel (2005). The assay is based on the oxidation of ferrous to ferric ion in acidic medium in the presence of various oxidant species, and the detection of ferric ion by xylenol orange. Calibration and construction of a standard curve were performed using a hydrogen peroxide concentration gradient (0–100 *μ*mol). The absorbance at 560 nm was measured on a spectrophotometer. The results were expressed as the mean micro molar equivalent of hydrogen peroxide per mg of tissue proteins [*μ*mol H_2_O_2_ Eq./mg proteins]^37^.

The total antioxidant status (TAS) was determined according to the method of Erel (2004). The method is based on decoloration of 2,2′-azinobis-(3-ethylbenzothiazoline-6-sulfonic acid radical cation [ABTS(*+)] by various antioxidants. Calibration and construction of standard curve were performed using a Trolox (6-hydroxy-2,5,7,8-tetramethylchroman-2-carboxylic acid) concentration gradient (0–100 *μ*M). The absorbance at 660 nm was measured spectrophotometrically. The results were expressed as the mean micromolar equivalents of Trolox per mg of tissue proteins [*μ*mol Trolox Eq./mg proteins]^38^.

The oxidative stress index (OSI) was defined as the ratio of TOS to TAS, and it was displayed by arbitrary unit = TOS [*μ*mol H_2_O_2_ Eq./mg proteins]/TAS [*μ*mol Trolox Eq./mg proteins]^38^.

The advanced oxidation protein products (AOPP) were determined spectrophotometrically at 340 nm and compared with a standard gradient solution of chloramine-T. The data were expressed as *μ*mol of chloramine-T equivalents per mg of proteins [*μ*mol Chloramine-T Eq./mg proteins]^39^.

### Data presentation and statistical analysis

All analysis was done by using Graph Pad Prism (Graph Pad Software Inc., San Diego, USA). The results were presented as mean ± SD of n observations. Statistical analysis was performed by using one-way analysis of variance (ANOVA) followed by Tukey post-hoc test. All calculated values were presented as mean ± SD. P-value lower than 0.05 was considered as statistically significant.
